# Complicated Invasive Pneumococcal Meningitis: Highlighting the Value of Implementing New Pneumococcal Vaccines

**DOI:** 10.7759/cureus.35482

**Published:** 2023-02-26

**Authors:** Stephen A Thacker

**Affiliations:** 1 Pediatrics, Medical University of South Carolina, Charleston, USA

**Keywords:** chatgpt, acute kidney injury, streptococcus sepsis, streptococcus pneumoniae meningitis, vaccine science and policy, meningitis, pneumococcal vaccine, infectious disease, invasive pneumococcal disease

## Abstract

Invasive *Streptococcus pneumoniae* disease (IPD) remains a serious cause of morbidity and loss of life in children and adults worldwide. While pneumococcal vaccines have reduced the frequency of invasive pneumococcal disease, the emergence of invasive non-vaccine serotypes has mandated the development of novel pneumococcal vaccines to further protect against these emerging serotypes. We present a case of a non-vaccine serotype invasive pneumococcal disease-causing septic shock, meningitis, and stroke in a previously healthy and appropriately vaccinated 23-month-old male.

## Introduction

As part of a journal-sponsored request for Artificial Intelligence (AI)-assisted case report collection, ChatGPT was extensively used to generate the following framework and text and identify some key references in this manuscript describing the invasive pneumococcal disease. Images of ChatGPT iterative engagement can be found in the Appendix.

Invasive pneumococcal disease (IPD) is a serious infection caused by the bacterium *Streptococcus pneumoniae *[1]. It can occur in both children and adults and can take the form of pneumonia, meningitis, or sepsis. In children, IPD is a major cause of morbidity and mortality worldwide. It is particularly prevalent in young children, those with underlying medical conditions, and those who are immunocompromised. The incidence of IPD in children has decreased in recent years due to the introduction of conjugate pneumococcal vaccines; however, it is still a significant public health issue [1-4].

Symptoms of IPD in children can include fever, cough, difficulty breathing, and lethargy. Diagnosis is typically made through a combination of clinical examination, laboratory tests, and imaging studies. Treatment usually involves antibiotics and supportive care but can also include more aggressive measures such as mechanical ventilation or extracorporeal membrane oxygenation (ECMO) in severe cases.

Preventive measures, such as vaccination and infection control, are important for reducing the incidence of IPD in children. The Centers for Disease Control and Prevention (CDC) recommends routine vaccination for all children against pneumococcal disease [5].

## Case presentation

**Figure 1:**
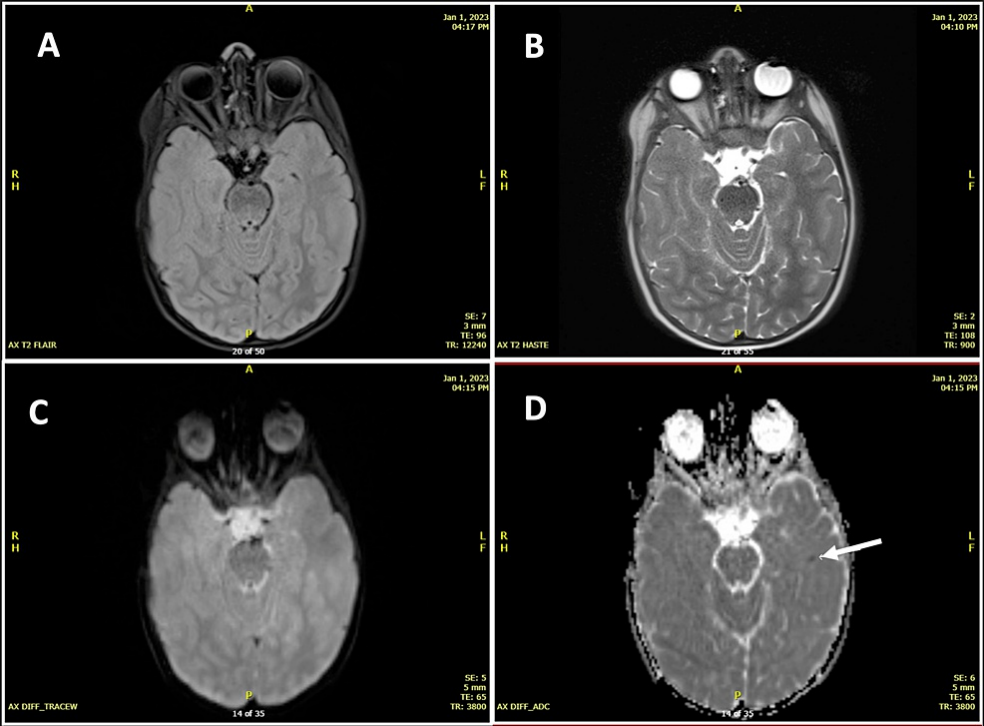
MRI brain without contrast. A. Axial T2 fluid-attenuated inversion recovery (FLAIR), B. Axial T2 half-Fourier acquisition single-shot turbo spin echo (HASTE), C. Axial trace diffusion (TRACEW), D. Axial apparent diffusion coefficient (ADC), Arrow denoting the punctate area of restricted diffusion in the left temporal lobe.

A 23-month-old boy with no significant past medical and fully immunized for age-based recommendations from the Advisory Council on Immunization Practices (ACIP) presented to a pediatric emergency department with 48 hours of intermittent fevers to a max of 103 degrees Fahrenheit, progressive irritability followed by increasing lethargy. The event followed a self-limited viral prodrome with rhinorrhea and congestion 10 days prior. There was no associated acute otitis media or clinical sinus disease. On presentation, he was found to be lethargic and demonstrating signs of meningismus and nuchal rigidity. Infectious workup included blood, urine, and central spinal fluid (CSF) sampling. Complete blood count (CBC) returned with 10,630 white blood cells/mm^3 ^with a differential of 73% neutrophils, 11% bands, 14% lymphocytes, hemoglobin of 10.3 gm/dL, and platelets of 432,000 cells/mm^3^. C-reactive protein (CRP) was 33.2 mg/dL; procalcitonin was 54.78 ng/mL. AST and ALT were 19 U/L and 11 U/L, respectively. Admission venous blood gas had a pH of 7.41, bicarbonate of 14.5 mmol/L, total CO2 of 13.5 mmol/L, O2 saturation of 91.9%, a base deficit of -8.9 mmol/L, an anion gap of 17.5. Blood culture obtained on admission grew *Streptococcus pneumoniae* within two hours of sampling. Urinalysis returned with a 3 WBC/high power field, negative nitrites, and leukocyte esterase, and his urine culture was negative. His CSF cell count was notable for 787 cells/mm^3^ (71% polymorphonuclear cells and 3% lymphocytes) with an elevated protein of 331 mg/dL and a CSF glucose of < 0.1 mg/dL with paired serum glucose of 115 mg/dL. CSF gram stain was positive for gram-positive cocci, and blood and CSF cultures ultimately returned positive for *Streptococcus pneumoniae *sensitive to cephalosporins with no clinically-relevant resistances. Initial empiric antibiotic therapy included vancomycin and ceftriaxone for broad bacterial meningitis coverage and acyclovir for antiviral coverage until the gram stain of the CSF returned positive. Steroids and mannitol were not given, but the patient did have strict fluid management for the possible syndrome of inappropriate antidiuretic hormone secretion (SIADH), which was resolved by hospital day two. Given the patient’s progressive lethargy and altered mental status, a magnetic resonance imaging (MRI) of the brain was obtained on hospital day two that demonstrated punctate areas of restricted diffusion in the left temporal lobe fitting with infarct, as shown in Figures 1A-1D. This infarct was not felt to be the sole etiology of his encephalopathic mental status changes but rather a sequelae of his meningitis. 

Electroencephalogram monitoring was negative for seizure activity but described global encephalopathy. The patient had a protracted critical illness course requiring three days of mechanical ventilation due to the inability to protect his airway and oliguric stage 3 acute kidney injury, with baseline creatinine (Cr) of 0.2 mg/dL rising to a peak of 1.8 mg/dL. The patient's AKI was multifactorial due to shock and the need for high-dose nephrotoxic antibiotics and antiviral and required continuous renal replacement therapy from hospital days three to 11 with the development of subsequent chronic kidney injury. The patient received two weeks of cephalosporin treatment with ceftriaxone or cefepime, based on the need for calcium-containing dialysate solution, for pneumococcal meningitis. The patient demonstrated steady clinical recovery and improvement in MRI findings of ischemia at the end of therapy. The small temporal lobe infarct was followed clinically without specific pharmacotherapeutic intervention but required speech, physical, and occupational therapy support. The patient required ongoing multispecialty support for chronic kidney disease and the sequelae of his sepsis and meningitis with nephrology, speech, and physical therapy care after discharge. Serotyping of the patient’s infecting *Streptococcus pneumoniae* returned as 10A.

## Discussion

Invasive pneumococcal disease (IPD) is a serious infection presenting in a myriad of diseases, including sepsis, pneumonia, complicated head and neck infection, and in its most severe form, meningitis. IPD is caused by the bacterium *Streptococcus pneumoniae* and can occur in both children and adults. This case highlights the potential complications of pediatric IPD, including meningitis, stroke, and overwhelming sepsis leading to multiorgan failure and lasting morbidity. Clinicians must remain informed and support the implementation of novel pneumococcal vaccine strategies to prevent both pediatric and adult IPD. 

Over the years, various pneumococcal conjugate vaccines (PCVs) have been developed to prevent IPD in children and adults, including PCV7, PCV13, PCV15, and PCV20. Additionally, one pneumococcal polysaccharide vaccine, PPSV23, continues to be licensed for use in the U.S. for those aged two years and older with risk factors and those 65 years and older. Each of these vaccines has a different composition and covers a different number of serotypes. PPSV 23 has not been shown to reduce bacterial carriage of vaccine serotypes, while all PCV vaccines have demonstrated a reduction in bacterial carriage of included serotypes [6,7]. While a significant reduction in vaccine serotype pneumococcal carriage has decreased following the introduction of pneumococcal conjugate vaccines, it has been recognized that non-vaccine serotypes increase in a nasal carriage over time [8]. 

PCV7 was the first pneumococcal vaccine introduced in 2000 and covered seven serotypes (serotypes 4, 6B, 9V, 14, 18C, 19F, and 23F) of pneumococcal bacteria. The impact of PCV7 was notable, with an associated significant reduction in IPD among children with upwards of 97% reduction in IPD due to vaccine serotypes [1].

PCV13, introduced in 2010, expanded the coverage to 13 serotypes (additional serotypes 1, 3, 5, 6A, 7F, 19A) (Table 1) and provided wider protection against IPD compared to PCV7. A recent study by Baxter et al. demonstrated that the use of PCV13 as part of routine childhood vaccine series was associated with a significant reduction in IPD among children, improving upon the reduction of IPD offered by PCV7 [2]. PCV15, introduced in 2021, covers 15 serotypes and is designed to provide wider coverage against IPD compared to PCV13. PCV15 has demonstrated safety, tolerability, and immunogenicity on par with prior pneumococcal conjugate vaccines [3]. PCV20, the latest pneumococcal vaccine introduced in 2021, covers 20 serotypes.

**Table 1:** Currently, U.S.-licensed pneumococcal vaccines included serotypes *Pneumococcal conjugate vaccine (PCV), **Pneumococcal polysaccharide vaccine (PPSV)

Vaccine	1	2	3	4	5	6A	6B	7F	8	9N	9V	10A	11A	12F	14	15B	17F	18C	19A	19F	20	22F	23F	33F
PCV*13	x		x	x	x	x	x	x			x				x			x	x	x			x	
PCV*15	x		x	x	x	x	x	x			x				x			x	x	x		x	x	x
PCV*20	x		x	x	x	x	x	x	x		x	x	x	x	x	x		x	x	x		x	x	x
PPSV**23	x	x	x	x	x		x	x	x	x	x	x	x	x	x	x	x	x		x	x	x	x	x

(1, 3, 4, 5, 6A, 6B, 7F, 8, 9V, 10A, 11A, 12F, 14, 15B, 18C, 19A, 19F, 22F, 23F, and 33F) and is designed to provide the widest coverage against IPD compared to earlier vaccines (Table 1). The potential impact of PCV20 on IPD among children is not yet clear, but early studies suggest that it may provide significant protection against IPD.

Current recommendations for routine vaccination for children include PCV13 or PCV 15 given to all infants as a primary series at ages two, four, and six months with a booster at ages 12 to 15 months. Additionally, children 2-18 years who are at the highest risk for serious pneumococcal infection are recommended to receive 1-2 PPSV23 vaccinations following completion of their PCV13 or PCV15 series [4]. While PCV20 is currently recommended for those 18 years of age and older, a recent phase 2 study found PCV20 safe, tolerable, and with similar immunogenicity to PCV13 [5].

Updated recommendations for pneumococcal vaccination include the use of PCV15 or PCV20 in all adults 65 years or older. If PCV15 is used, a single dose of PPSV23 is recommended one year later. If PCV20 is given, an additional dose of PPSV23 is not recommended. People aged 19 through 64 years with high risk for pneumococcal disease who have no history of pneumococcal vaccination or an unknown pneumococcal vaccination history should receive either a single dose of PCV20 alone or a dose of PCV15 followed by a dose of PPSV23 at least one year later [4].

## Conclusions

This case highlights the need for continued surveillance and expansion of pneumococcal serotypes to continue to minimize the impact of invasive pneumococcal disease. The 10A serotype that caused the devastating disease in this child is currently covered in PCV20. While current recommendations do not include the use of PCV20 in those under aged 18 years, a recent phase 2 study found PCV20 safe, tolerable, and with similar immunogenicity to PCV13. This case highlights the potential enhanced mitigation against invasive pneumococcal disease in children PCV20 could have if licensed for children in addition to the proven benefit for adults. Pediatric and adult clinicians should be aware of the recently updated pneumococcal vaccination recommendations to ensure they receive the appropriate vaccine for their age and health status.

## Figures and Tables

**Figure 2:**
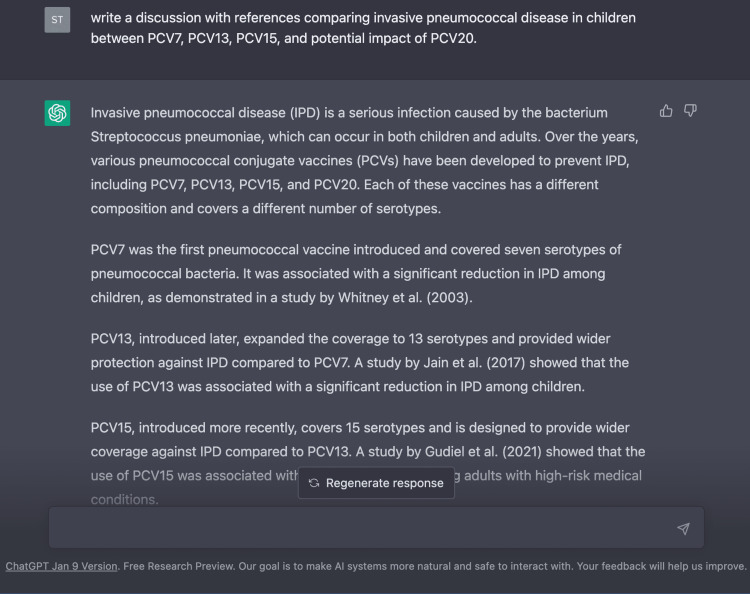
ChatGPT Thread A

**Figure 3:**
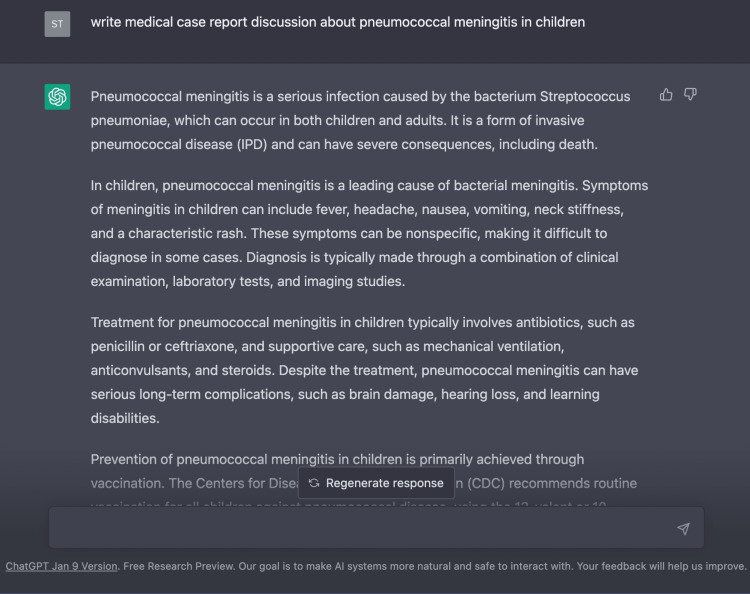
ChatGPT Thread B

## Appendices

Screenshots of engagement with ChatGPT

## References

[1] Whitney CG, Farley MM, Hadler J (2003). Decline in invasive pneumococcal disease after the introduction of protein-polysaccharide conjugate vaccine. N Engl J Med.

[2] Baxter R, Aukes L, Pelton SI (2021). Impact of the 13-valent pneumococcal conjugate vaccine on invasive pneumococcal disease after introduction into routine pediatric use. J Pediatric Infect Dis Soc.

[3] Stacey HL, Rosen J, Peterson JT (2019). Safety and immunogenicity of 15-valent pneumococcal conjugate vaccine (PCV-15) compared to PCV-13 in healthy older adults. Hum Vaccin Immunother.

[4] Kobayashi M, Farrar JL, Gierke R (2022). Use of 15-valent pneumococcal conjugate vaccine among U.S. children: updated recommendations of the Advisory Committee on Immunization Practices — United States, 2022. MMWR Morb Mortal Wkly Rep.

[5] Senders S, Klein NP, Lamberth E (2021). Safety and immunogenicity of a 20-valent pneumococcal conjugate vaccine in healthy infants in the United States. Pediatr Infect Dis J.

[6] van Deursen AM, van Houten MA, Webber C (2018). The impact of the 13-valent pneumococcal conjugate vaccine on pneumococcal carriage in the Community Acquired Pneumonia Immunization Trial in Adults (CAPiTA) Study. Clin Infect Dis.

[7] Adler H, German EL, Mitsi E (2021). Experimental human pneumococcal colonization in older adults is feasible and safe, not immunogenic. Am J Respir Crit Care Med.

[8] Mbelle N, Huebner RE, Wasas AD, Kimura A, Chang I, Klugman KP (1999). Immunogenicity and impact on nasopharyngeal carriage of a nonavalent pneumococcal conjugate vaccine. J Infect Dis.

